# Quantifying the Fitness Advantage of Polymerase Substitutions in Influenza A/H7N9 Viruses during Adaptation to Humans

**DOI:** 10.1371/journal.pone.0076047

**Published:** 2013-09-27

**Authors:** Judith M. Fonville, David F. Burke, Nicola S. Lewis, Leah C. Katzelnick, Colin A. Russell

**Affiliations:** Department of Zoology, University of Cambridge, Cambridge, United Kingdom; University of Edinburgh, United Kingdom

## Abstract

Adaptation of zoonotic influenza viruses towards efficient human-to-human transmissibility is a substantial public health concern. The recently emerged A/H7N9 influenza viruses in China provide an opportunity for quantitative studies of host-adaptation, as human-adaptive substitutions in the PB2 gene of the virus have been found in all sequenced human strains, while these substitutions have not been detected in any non-human A/H7N9 sequences. Given the currently available information, this observation suggests that the human-adaptive PB2 substitution might confer a fitness advantage to the virus in these human hosts that allows it to rise to proportions detectable by consensus sequencing over the course of a single human infection. We use a mathematical model of within-host virus evolution to estimate the fitness advantage required for a substitution to reach predominance in a single infection as a function of the duration of infection and the fraction of mutant present in the virus population that initially infects a human. The modeling results provide an estimate of the lower bound for the fitness advantage of this adaptive substitution in the currently sequenced A/H7N9 viruses. This framework can be more generally used to quantitatively estimate fitness advantages of adaptive substitutions based on the within-host prevalence of mutations. Such estimates are critical for models of cross-species transmission and host-adaptation of influenza virus infections.

## Introduction

The emergence of novel influenza A viruses in humans is an ongoing cause for concern. The outbreak of human infections of A/H7N9 influenza viruses in China in early 2013 highlights the potential for emergence of influenza viruses not previously detected in humans [1]. The animal source of the A/H7N9 infections has not yet been unambiguously confirmed, although highly similar viruses have been isolated from birds in live bird markets epidemiologically linked to human infections [2], [3]. Analogous to the situation for other zoonotic influenza viruses such as A/H5N1, it is feared that adaptation of A/H7N9 viruses could lead to increased incidence of human infection and sustained human-to-human transmission. Information on the potential of such zoonotic viruses to become adapted for efficient transmission between humans is of critical importance in decisions on eradication and pandemic mitigation strategies. However, studies on within-host evolution of pathogens are often limited by sparse information on the magnitude of the selective advantage that substitutions might have [4].

Several substitutions in the influenza virus polymerase PB2 gene have been reported to be important for adaptation of avian influenza viruses to human infection [5]–[18]. For example, E627K, D701N, and Q591K have been shown to alter the host range of the virus, in various subtypes including A/H2N2, A/H5N1 and A/H7N7, as they increase virulence, replication and pathogenicity in mammalian cells [5]–[18], and the E627K substitution has been reported to increase the virulence of the novel A/H7N9 strains in mice [19]. The effects of the PB2 substitutions may be the result of altered temperature-dependence of polymerase activity [15], [20], [21], yet are dependent on the viral genetic backbone and the cell type or model animal used [8], [16], [21]. Interestingly, substitutions E627K and D701N are also reported to be determinants of mammalian inter-host transmission [15], [22]. Both substitutions can improve the efficiency of influenza virus growth in the human respiratory tract. The mutation Q591K has been reported to increase the polymerase activity and pathogenicity of avian influenza A/H9N2 viruses, and to allow efficient replication of A/H5N1 and A/H1N1pdm viruses in mammals [23]–[25]. Several studies have proposed that the absence of one PB2 substitution can be compensated for by a substitution at another position [13], [22], [23]. Importantly, E to K, D to N, and Q to K substitutions can always be achieved through a single nucleotide mutation.

Published reports of PB2 sequence data from the 2013 A/H7N9 virus outbreak in China highlight that the PB2 genes from birds and environmental samples (40 of 40) have 627E, 701D, and 591Q, whereas the PB2 genes of the A/H7N9 viruses collected from humans have acquired either the E627K (11 out of 13), the D701N substitution (1 out of 13), or the Q591K substitution (1 out of 13) [1]–[3], [19], [26]–[29]. Thus, based on the currently available data, which are biased towards severe infection cases as no sequences of subclinical infections are available, all sequenced viruses from human A/H7N9 infections (13 of 13) show signs of human adaptation.

Despite the evidence for airborne transmissibility of the A/H7N9 virus among ferrets and ability to infect non-human primates and pigs [19], [30]–[32], epidemiological and genetic analyses have so far not provided evidence for sustained human-to-human transmission, suggesting that the human A/H7N9 infections are the result of multiple independent zoonotic introductions [1], [26], [33]. Indeed, the general lack of secondary infections among individuals in close contact with infected persons [33], [34] and the genetic divergence between viruses isolated from humans [26] suggest that widespread circulation of A/H7N9 viruses among non-human hosts must have occurred in China [35]. Thus, a critical observation is that adaptive PB2 substitutions have been observed within all humans from whom virus was isolated and sequenced. As suggested by Jonges *et al*. [26], and similar to the situation of drug-resistance in HIV [36], two mechanisms could have lead to the detection of such adaptive substitutions in a single host after each of these cross-species transmission events: 1) the substitution was acquired during replication within the human host [2], [26]; or 2) the virus population infecting the human already contained one or more virions with the adaptive substitution at a sufficiently low proportion that they would not have been detected by consensus sequencing in the avian host. In order to reach a within-human prevalence detectable by consensus sequencing, these absent or initially rare substitutions would need a selective advantage to substantially increase in proportion. Here, we employ the probabilistic mathematical framework described in Russell *et al*. [4] to quantify the selective advantage necessary to observe a substitution in the majority of virions within a human host over the course of a single infection.

## Results

We explored the minimum selective advantage that an adaptive mutant must have compared to the wild type virus in order to reach a certain within-host proportion as a function of duration of infection or the time point of infection at which the sequenced virus was collected. For the model used here, fitness advantage is defined as a replicative fitness advantage, i.e. the relative change in progeny of the mutant with respect to progeny of the starting virus (i.e. the wild type virus is assumed to have a neutral fitness: fitness 1). Any fitness advantage of the mutant is exercised in each step of replication. Results are given for a polymerase error rate, *r*, of 10^−5^ mutations per site per round of replication, and the results for *r* = 10^−4^ and 10^−6^ are shown in brackets.

**Figure 1:**
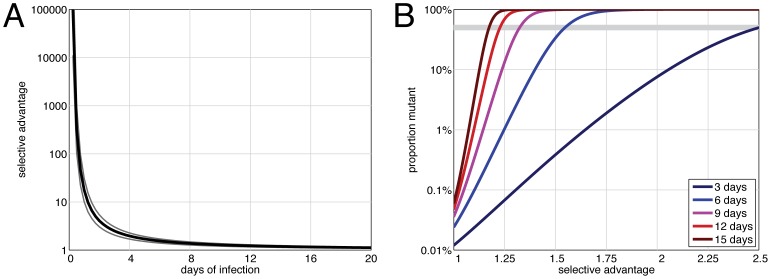
Selective advantages when the substitution is absent at the start of infection. A) The selective advantage required to achieve 50% (black) prevalence of the adaptive substitution within the host after a certain number of days of infection in a situation where the substitution is not present in the infecting virus population (results for 10% and 90% shown in grey). B) The proportion of mutant observed after 3 (dark blue), 6 (blue), 9 (pink), 12 (red) and 15 (dark red) days of infection for a range of selective advantages. The grey bar indicates 50% prevalence.

Figure 1A shows the minimum fitness advantage required to achieve a virus population where at least 10%, 50% or 90% of the virions have a specific mutation, in the case that the mutation was absent at the start of infection. This figure only establishes a lower bound for the required fitness advantage – any higher fitness advantage would also reach or exceed the given proportion. While extreme fitness advantages are required to achieve a given proportion in the first day or two of infection, the required fitness advantage decreases rapidly as the length of infection increases. A selective advantage of 1.55 (1.39 for *r* = 10^−4^, 1.71 for *r* = 10^−6^) is sufficient to reach 50% adaptive-mutant prevalence after 6 days of infection, a substantial decrease in the lower bound on the selective advantage required compared to the situation after only 2 days of infection, which would require an advantage of at least 4.07 (3.01, 5.48). The differences in selective advantage required to achieve 10%, 50% and 90% prevalence appear relatively small (Figure 1A) due to the exponential growth of mutant proportions and the fact that the difference between 10% and 90% is less than an order of magnitude.

The proportion of viruses with an adaptive substitution after 3, 6, 9, 12 and 15 days of infection as a function of the selective advantage of the adaptive substitution is shown in Figure 1B. If there is no selective advantage, i.e. the mutant has the same fitness as the wild type virus, the proportion of adapted mutant after 6 days is predicted to be 0.024% (0.24%, 0.0024%). As also shown in Figure 1A, the longer the infection, the lower the selective advantage needed to achieve any given proportion. As expected, for a given length of infection, the higher the selective advantage, the higher the proportion of mutant.

**Figure 2:**
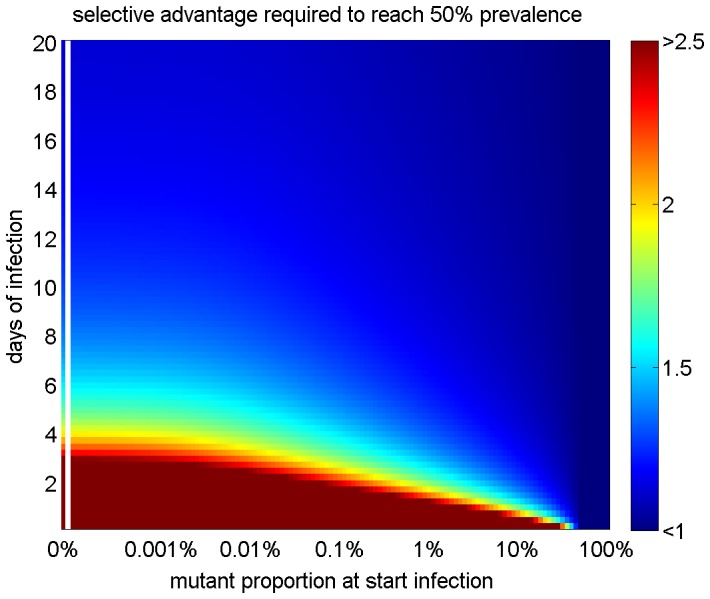
Quantifying the selective advantage as a function of substitution prevalence at the start of infection and infection duration. The minimum selective advantage necessary to reach 50% prevalence in the human host is displayed in color as a function of days of infection (y-axis) and mutant proportion at start of infection (x-axis). Selective advantages of 2.5 and 1 represent the upper and lower bounds of the color bar. The selective advantage exceeds 2.5 for early time points (see Figure 1A), but for clarity, a threshold was used here. For starting prevalences of >50% no selective advantage is required.

Even though the E627K, D701N, and Q591K substitutions have not been reported for non-human A/H7N9 viruses, it is not possible to rule out that these substitutions were present as part of the viral diversity in the non-human host species. The absence of the PB2 substitutions in the non-human sequences may be because only the predominant nucleotide at each position in the virus sample is reported in the consensus sequences. Figure 2 shows the selective advantage required for a substitution to increase to 50% prevalence in the virus population in a human host as a function of the fraction of mutant present at the start of infection and the time since infection. The presence of the mutant at the start of infection decreases the fitness advantage needed for a mutant to reach predominance. A virus population that has 10% mutant at the start of infection requires a fitness advantage of 1.10 to reach 50% after 6 days, compared to 1.55 for 0% presence at start of infection. As expected, the required fitness advantage decreases as the length of infection increases, because the fitness benefit is expressed over more generations, and thus smaller fitness advantages can still result in a substitution reaching predominance (see also Figure 1B). Indeed, for long infections, the substitution needs a relatively small fitness advantage over the wild type to achieve 50% prevalence (e.g. 1.12 (1.09, 1.16) to be predominant after 20 days of infection), even if the mutant was completely absent at the start of infection.

In publically available clinical data, the shortest reported duration between the onset of disease symptoms and the sampling for A/H7N9 sequencing was 6 days [1], [2]. If this presumed incubation time, which may have to be adjusted with any amount of time between the start of infection and the time of symptom onset, which is currently unknown, corresponds to the length of time during which the virus was replicating, the substitution would require a fitness advantage of 1.33 (1.31, 1.33) to achieve 50% prevalence if the mutant was already present at 0.1% when the infection started and 1.55 (1.39, 1.71) if the mutant was absent at the start of infection.

## Discussion

In general, there is a lack of quantitative information on fitness effects of genetic substitutions in influenza viruses. The A/H7N9 outbreak offers an opportunity to calculate a within-host fitness advantage in humans, which circumvents the need to rely on estimates from *in vitro* studies. Our approach uses the time since the start of infection to establish a lower bound on the within-host fitness advantage of the human-adaptive PB2 substitutions in A/H7N9. Such information on the fitness effects of adaptive substitutions fills a gap in our knowledge when modeling within-host evolution and host-adaptation of viruses, and this modeling framework can be used to obtain fitness advantage information for other situations in which the proportion of adapted virus in the human host after a certain time is known as a function of the proportion of adaptive mutant at the start of infection. Sensitive and accurate estimates of low-prevalence mutations from deep sequencing would allow the estimation of fitness advantages of less strongly selected mutations and help expand the applicability of the framework to situations where the mutant would not have been detected with consensus sequencing alone.

It is likely that the genetic background of the virus in which these substitutions occur modulated the observed pattern of PB2 substitutions in the non-human and human A/H7N9 strains. For example, the E627K and D701N substitutions have only been detected in ∼30% and ∼7%, respectively, of sequences from human infections with A/H5N1 viruses and in ∼23% and <1% of sequences from avian infections with A/H5N1 viruses [4], [37]. For A/H5N1 viruses, the Q591K substitution has not been observed in viruses from birds and has been found in <1% of human infections. The majority of sequenced A/H5N1 viruses from birds that have the E627K substitution are phylogenetically grouped in clade 2.2 and its subclades with ∼91% of viruses having the substitution, compared to ∼1% prevalence detected in sequences from other clades. The E627K substitution is found in all (12/12) sequenced A/H5N1 viruses from clade 2.2 and its subclades isolated from humans, the prevalence of the substitution for all other clades of A/H5N1 viruses from humans is ∼27%. For A/H9N2 viruses, E627K, D701N, and Q591K have been found in 0%, 10% (1 of 10 sequences), and 0% of the human infections and in <1%, 0%, <1% of the reported avian data. This variability in prevalence indicates that the fitness advantage that any specific substitution confers differs among influenza subtypes and constellations of internal genes, and more detailed analyses on the influence of genetic background are needed.

The differences in adaptive substitution prevalence among subtypes may be the result of the possibility to obtain various functionally equivalent adaptive substitutions, and different substitutions may be preferentially associated with each subtype. A wide range of potential mammalian-advantageous mutations in PB2, in addition to E627K, D701N and Q591K, has been reported in the literature [10]–[12]. In the absence of other similar studies, the A/H7N9 data provide the current best estimate for the fitness associated with this mutation in the PB2 gene. Because the fitness advantage is defined relative to the wild type virus, an equivalent way to interpret these data is that they provide a measure of how unfit the starting A/H7N9 virus was compared to the PB2-adapted virus in humans. Other wild type viruses might be more relatively fit than this A/H7N9 virus was in humans in which case the selective advantage of adaptive substitutions in the PB2 gene would be smaller.

The E627K substitution was found in one human case out of the 61 sequenced patient strains from the A/H7N7 influenza outbreak in 2003 in the Netherlands [38], [39]. In this fatal infection, the patient presented with pneumonia in combination with acute respiratory stress syndrome [38], instead of the conjunctivitis symptoms mostly observed during the A/H7N7 outbreak. Although D701N circulated in some A/H7N7 viruses from poultry during that outbreak [39], the E627K PB2 substitution is thought to have arisen during replication of the virus in the human patient, as it was not observed in any isolates from poultry or other humans [8], [26], [38], [39]. The pathogenicity of the A/H7N7 virus isolated from this patient is thought to be associated with the E627K substitution [6], similar to the effect of this substitution in A/H5N1 [14]. To our knowledge, this is the only previously well-documented situation in which there is a time frame for an adaptive PB2 mutation to arise during the infection of a *single* human host.

The possibility that adaptive PB2 substitutions in A/H7N9 viruses may exist at a high prevalence in a non-human host cannot be fully dismissed: we cannot exclude that the A/H7N9 viruses sequenced so far are simply not representative of viruses in the poultry that infected the human cases, or that the animal source from which human infection arose is not amongst the species that have been identified and sequenced so far. However, the viruses sequenced from humans are highly similar to the viruses isolated from avian and environmental samples, making this possibility unlikely. Another possibility is that the substitutions have arisen during laboratory virus culturing, as seasonal influenza virus isolates from humans are commonly passaged in MDCK cultures, whereas avian viruses are often passaged in eggs, prior to sequencing. However, for the A/H7N9 viruses sequenced thus far, the majority of human and avian isolates have been cultured in eggs, and thus this explanation is not sufficient for the observed presence of PB2 substitutions in human isolates.

The human infection samples from which sequences have been collected and made available in Genbank and from published reports [1]–[3], [28], [34], [40] may not reflect the within-host evolutionary dynamics of all human infections. From the reported data it is clear that co-morbidities such as detection of hepatitis B antigen or hypertension are common, but not present for all individuals from whom sequences have been obtained; this is similar to the gross epidemiology of all reported A/H7N9 infections, where 68% of the individuals have a reported coexisting condition, often hypertension [41]. Similarly, for the sequences from individuals for which treatment details are known, oseltamivir or other antivirals are administered in some, but not all cases, and in some cases only after the sample for sequencing was already obtained. In one analysis of 111 reported H7N9 cases, 108 individuals received antivirals, yet for 65 of these 108, treatment was only initiated at or after 6 days post-symptom onset [41]. Although there are substantial unknowns about the potential bias of reporting A/H7N9 infections [42], and any biases in selection of samples for sequencing, there is currently no reason to assume that these patterns in PB2 substitutions are the result of phenomena or underlying factors that are not representative of all individuals reported with A/H7N9 infections.

The results presented here provide a lower bound on the selective advantage of the PB2 adaptive substitutions in A/H7N9 per genome replication round, as a function of the duration of infection at the time of virus sample collection. The exact duration of time between symptom onset and swab varies from 6 to 22 days in the literature for the available sequences [1]–[3], [28], [34], [40], and the model can be adjusted to report the lower limit for that individual based on their data. The longer the infection, the lower the estimated lower bound, which is why it is important to obtain the sequence information at the earliest possible time point. If samples collected at earlier time points in infection than those reported in the literature so far reveal a predominance of an adaptive substitution, the fitness advantage can be adjusted upwards, based on the information in Figures 1 and 2; similarly the timing has to be adjusted with information on how long viral replication is occurring before symptom onset, given that often the exact time of infection is unknown.

If no adaptive substitutions are detected in a newly sequenced human virus, there are several potential explanations for such an observation: either the virus was sampled before the substitution could reach the threshold for detection (e.g. due to short time of infection, low presence of mutant at start of infection, or stochastic effects), or host-specific factors altered the viral population dynamics, or the genetic background of the virus affected the selective advantage of the substitution. If the reported sequences are each the result of stuttering chains of human-to-human transmission of the A/H7N9 virus, potentially undetected if A/H7N9 viruses without a PB2 substitution do not cause symptomatic disease, the effective within-human-host evolutionary time may be significantly longer than for a single infection, and the fitness boundary could be substantially lower, depending on the size of the transmission bottleneck and the selection of adapted virions during transmission [4]. Indeed, if absence of the adaptive PB2 substitutions would cause asymptomatic or low-pathogenic infections, this could have caused an observational bias where the only sequences are from severe infection cases which have the PB2 substitutions, and thus would lead to overestimation of the general fitness advantage of such a substitution. To test this bias, it would be important to sequence the viruses isolated from mild A/H7N9 influenza infections [42], and if this indicates a difference in PB2 substitution prevalence, seroprevalence percentages for individuals with extensive contact with non-human hosts would help to assess the size of this bias in our data. On the other hand, if it is found that one of the PB2 substitutions is necessary to even initiate a human infection (i.e. with a fully purifying selective advantage, if selective advantage is defined at the transmission step), the lower bound of the within-host fitness advantage of the PB2 substitution can only be estimated as neutral (fitness 1) as it would be the sole component of the within-human viral diversity.

The situation for A/H7N9 within-host evolution of PB2 substitutions is akin to the situation of drug resistance, where drug-resistant mutants rapidly rise, either due to pre-treatment low-level prevalence, or *de novo* generation [36]. Our modeling framework could also be used in this situation, and indeed, if highly sensitive deep sequencing data are available, also to study any fitness disadvantage that such resistant mutations might have in the absence of treatment. This information could then be used in models such as used by Ribeiro *et al*. to investigate the production of resistant HIV mutants, and optimize timing of antiretroviral therapy [43]; similar studies could be carried out to parameterize models of antiviral treatment in influenza infection.

The strong-selection-weak-mutation paradigm [44], [45] has been employed for models of within-host evolution, and assumes that advantageous mutations go to fixation faster than new mutations arise, through applying a strong selection on beneficial mutations. Given the high mutation rates of RNA viruses, the validity of such assumptions will critically depend on how beneficial a substitution is (e.g. a fitness advantage of 10 vs. 1.001), hence the framework reported in this study will be useful to determine weather a particular mutation is strongly or weakly selected, which will be informative for testing such assumptions. Quantifying and comparing the fitness effect of various host-adaptive substitutions can inform future qualitative and quantitative models of within-host evolution and cross-species transmission events. Accurate estimates of fitness parameters for adaptive substitutions are not only important for basic research, but may also be used to enhance quantitative risk assessments and to design virus control and pandemic preparedness strategies.

## Methods

The within-host population dynamics of virus mutants were calculated based on the deterministic probability model described in Russell *et al*. [4]. Errors made by the virus polymerase are the source of mutation: the probability of obtaining the advantageous nucleotide change, e.g. resulting in E627K, is 10^−5^ per virus genome per round of genome replication. There are two rounds of genome replication within each infected cell: one from vRNA to cRNA and one from cRNA to vRNA. Rounds of replication are transformed into time by assuming that each step of genome replication lasts six hours (e.g. 3 days corresponds to 12 rounds of genome replication, and 6 cellular lifecycles). Extensive details and discussion of the model and its parameterization were provided in the supporting material of Russell *et al*. [4]. All models and figures were created with Matlab R2012b (The Mathworks, MA, USA).

The population of each mutant type (viruses with and without mutation) *N_j_* after a replication cycle is given by the sum of contributions from each type in the previous replication cycles:
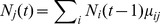
(1)


Where *μ_ij_* is the probability of type *i* mutating to type *j* (similar to e.g. Ribeiro *et al*. [36] and Perelson *et al*. [46]); each type contributes exactly its expected value. To calculate μ, the possibility of back mutation is incorporated (which does not significantly alter the results, but is a small conceptual improvement to the model presented by Russell *et al*. [4]). The probability of no mutation (μ_00_ and μ_11_) is *1-r*, while the probability of mutation (μ_01_ and μ_10_) is *r*.

The fitness advantage was modeled by adjusting the expected probability for a mutant at the end of each generation with an iterative algorithm. The advantage or disadvantage of each mutant type was expressed in each genome replication step: after every replication round, the relative progeny of the mutant and wild type viruses are adjusted based on their fitness values (thus, in effect, an advantageous substitution is equivalent to the wild type being disadvantageous). The starting population (generation zero) consists of N_0_ = 1 and N_1_ = 0 for Figure 1, these values are adjusted accordingly in Figure 2 to reflect the percentage of mutant (*q*) at the start of infection: N_0_ = 1−q and N_1_ = q. After application of the mutation matrix in equation 1, the probability of each type *N_j_* is multiplied by its relative fitness *g_i_*, and the population is normalized by dividing each type by the sum of the fitness-weighted prevalence of all types:
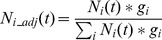
(2)


The *N_i_adj_* are then used as *N_i_* in equation 1 in the next replication step. The *fminsearch* algorithm (Matlab) was used to find at which settings the population of mutants first exceeded 50%, 10% or 90% as indicated.

This deterministic model reports the probability that any random virion of the total virus population has the mutation of interest. This probability is the same for each virion, and is insensitive to the virus population size and dynamics [4]: it is the merely monitoring the probability (equivalent to the proportion) of obtaining a given mutation, as a function of time.

In the model, we assumed that the fitness effect of the substitution in PB2 is observed in every replication step, as the polymerase is involved in each step. However, the methodology can easily be adjusted for other adaptive substitutions (such as in the HA gene) that would be only expressed at a subset of time points. The same methodology can be applied to estimate the fitness (dis)advantages of other adaptations, or for mutations in a different genetic backbone. The model does not incorporate any effects the immune response may have on the accumulation of mutations. However, if the immune response would not differentially neutralize the wild type and mutant virions, then the reported proportions and results would not be affected. Finally, the polymerase error rate *r* can be adjusted if the estimate of 10^−5^ used here, based on information for other influenza viruses, is not representative for A/H7N9 viruses (results for *r* = 10^−4^ and *r* = 10^−6^ are given in the text, by adjusting the values in the mutation matrix given in equation 1 accordingly).
